# Association between Cardiometabolic Index (CMI) with muscle mass and strength in US adults: A cross-sectional study

**DOI:** 10.1097/MD.0000000000046128

**Published:** 2025-11-21

**Authors:** Yuwen Shangguan, Shiqi Yu, Young-Je Sim, Kunyi Huang, Han Yuan, Yang Wang, Chen Chen, Zhenhao Lin, Zining Zhu

**Affiliations:** a Department of Exercise Physiology, Kunsan National University, Gunsan, Jeollabuk-do, South Korea; b School of Sports and Health, Shanghai University of Sport, Shanghai, China; c Department of Health and Physical Education, The Education University of Hong Kong, Tai Po, Hong Kong; d School of Sports and Health, Shandong Sport University, Jinan, Shandong, China; e College of Sports and Health, Harbin Sport University, Haerbin, China.

**Keywords:** CMI, cross-sectional study, grip strength, muscle mass, NHANES

## Abstract

Cardiometabolic Index (CMI) is a comprehensive indicator of lipid metabolism and visceral fat distribution, but its relationship with skeletal muscle mass and muscle strength remains unclear. We therefore explored the relationship between CMI and muscle health, providing new insights into the overall assessment of metabolic health. This cross-sectional study analyzed data from the National Health and Nutrition Examination Survey 2011–2014 cycles, including 2719 adults aged ≥ 18 years. During the study, participants’ Appendicular Skeletal Muscle Mass Index (ASMI) was determined using dual-energy X-ray absorptiometry, while their grip strength was measured by applying a dynamometer. CMI was calculated as triglycerides divided by high-density lipoprotein cholesterol, multiplied by waist-to-height ratio. Models were adjusted for age, sex, race/ethnicity, education, body mass index (BMI), lifestyle factors, and comorbidities. The correlation between CMI and skeletal muscle mass and grip strength was explored through weighted generalized linear regression modeling. Restricted cubic spline models were used to explore nonlinear associations and threshold effects. Further subgroup analyses were performed to clarify the moderating role of factors such as gender, ethnicity, and BMI in these relationships. CMI showed a significant positive correlation with ASMI (fully adjusted model: β = 1.01, 95% confidence intervals: 0.86–1.17, *P* < .0001) and showed a nonlinear relationship: at lower levels of CMI (<0.6), the strongest effect of CMI on ASMI was observed (β = 5.09); at higher levels (>0.6), this effect was significantly weaker (β = 1.46). Preliminary models of CMI and grip strength showed a positive correlation (Model 1: β = 2.74, 95% confidence intervals: 2.02–3.46, *P* < .0001), but this correlation weakened and became nonsignificant in the fully adjusted model. Subgroup analyses indicated that gender, race, and body mass index significantly moderated the relationship between CMI and ASMI, with females, non-Hispanic blacks, and high BMI being more strongly associated with ASMI. CMI is significantly positively correlated with skeletal muscle mass, and this association exhibits nonlinear and threshold effects. The relationship between CMI and muscle strength is more complex and may be influenced by other confounding factors.

## 1. Introduction

Cardiometabolic Index (CMI) is an emerging composite measure of metabolic health, calculated from triglycerides (TG), high-density lipoprotein cholesterol (HDL-C), and waist-to-height ratio (WHtR).^[1]^ Compared to traditional single metabolic indicators, CMI provides a more comprehensive reflection of an individual’s lipid metabolism, visceral fat accumulation, and cardiovascular metabolic status.^[2]^ In recent years, CMI has been widely used to assess the risk of obesity,^[3]^ insulin resistance, diabetes,^[4]^ and cardiovascular diseases (CVD).^[5]^ However, the impact of CMI on skeletal muscle health has not been systematically explored. Skeletal muscle is not only the largest organ in the human body but also a key metabolic organ, playing an important role in energy metabolism, glucose regulation, and insulin sensitivity.^[6]^ Skeletal muscle mass and muscle strength are core indicators of skeletal muscle health, closely linked to quality of life, metabolic health, and the incidence of chronic diseases.^[7]^ Specifically, sarcopenia and the decline in muscle function are considered independent risk factors for increased all-cause mortality during aging.^[8]^ Therefore, investigating the relationship between the metabolic indicator CMI and skeletal muscle health is crucial for understanding the impact of metabolic disorders on overall health.

Existing studies have confirmed that metabolic syndrome-related indicators (such as obesity, insulin resistance, and fatty liver) may negatively affect skeletal muscle function through inflammation, oxidative stress, and insulin signaling pathways.^[9,10]^ Furthermore, the relationship between metabolic syndrome and skeletal muscle may be bidirectional: metabolic disorders lead to a decline in muscle mass and function, while reduced muscle mass may further worsen metabolic health.^[11]^ While CMI has been extensively studied in relation to cardiovascular and metabolic diseases, its association with skeletal muscle strength remains underexplored. Given that muscle strength is a crucial indicator of aging-related decline, clarifying its relationship with CMI may reveal shared metabolic and inflammatory pathways linking cardiometabolic conditions and sarcopenia. However, despite being a comprehensive indicator of cardiometabolic health, there are still relatively few studies on the relationship between CMI and skeletal muscle mass and strength. Additionally, existing literature has primarily focused on the relationship between individual metabolic indicators (such as body mass index [BMI], TG, and waist circumference [WC]) and muscle health,^[12]^ with a lack of systematic analysis of CMI as a composite measure. More importantly, the relationship between metabolic health and skeletal muscle health may be moderated by various individual factors. Previous studies have found that sex, race, and body composition (such as BMI) play significant roles in moderating the relationship between metabolic health and muscle function. For example, women and obese individuals may exhibit stronger metabolic stress,^[13]^ while non-Hispanic Black individuals may have higher skeletal muscle mass but lower metabolic health risks.^[14]^ These differences suggest that research on the association between CMI and skeletal muscle health needs to account for the heterogeneity of individual factors.

Based on this, the present study utilized a cross-sectional analysis of National Health and Nutrition Examination Survey (NHANES) data from 2011 to 2014 to explore the association between CMI and Appendicular Skeletal Muscle Mass Index (ASMI) and grip strength, to provide a new research perspective for in-depth revelation of the complex mechanisms between metabolic health status and skeletal muscle function, and to provide a scientific basis for the development of targeted health interventions in the future.

## 2. Materials and methods

### 2.1. Study population

NHANES is a study that uses a complex sampling design to assess the nutritional and health status of the U.S. population. Data for the study were collected by visiting participants’ homes to conduct health-related questionnaire interviews, administering health physicals at mobile examination centers, and conducting laboratory biospecimen collection and testing. The research protocol for the survey was reviewed and approved by the Ethics Committee of the National Center for Health Statistics.

**Figure 1.**
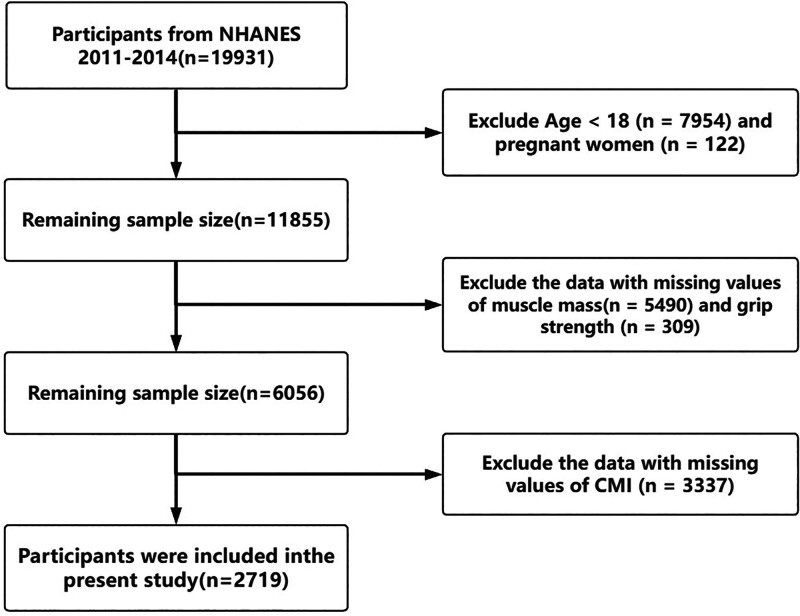
Flowchart of sample selection in NHANES 2011–2014. CMI = Cardiometabolic Index, NHANES = National Health and Nutrition Examination Survey.

The study initially included 19,931 participants (NHANES 2011–2014). Figure 1 illustrates the inclusion and exclusion process for our study. First, we excluded individuals under the age of 18 and pregnant women. Next, we excluded 5490 participants with missing appendicular lean mass (ALM) data and 309 participants with missing grip strength data. Then, we excluded 3337 participants who were missing data on height, WC, TG, and HDL-C, which were required to calculate CMI. Finally, our study included 2719 participants.

### 2.2. Calculation of CMI

WHtR was calculated as WHtR = WC (cm)/body height (BH; cm). CMI was calculated as CMI = TG (mmol/L)/HDL-C (mmol/L) × WHtR.

### 2.3. Assessment of skeletal muscle mass and hand grip strength

Dual-energy X-ray absorptiometry was used to assess skeletal muscle mass. Its advantages include low radiation dose, precise differentiation of tissue components, and high reproducibility. Exclusion criteria included pregnancy, use of radiographic contrast agents within the past 7 days, body weight exceeding 450 lb, or height over 6 ft 5 in. According to the European EWGSOP2 standards, muscle mass is body-size dependent, meaning larger individuals typically have greater muscle mass. Therefore, the ASMI was used as an index to assess muscle mass, which was calculated by dividing the value of appendicular lean body mass (ASM) by the square of the participant’s height (ASM/height²).^[15]^ According to the diagnostic criteria for sarcopenia, a loss of muscle mass is recognized when the ASMI is <7.0 kg/m² in men or 5.5 kg/m² in women.^[16]^

Grip strength was measured according to the guidelines in the Muscle Function Procedures Manual, using a Takei digital handgrip dynamometer (model T.K.K.5401, Niigata, Japan). Before the measurement, the device was adjusted to fit the participant’s hand size. During testing, participants stood with their arms fully extended and wrists in a neutral position. Maximum grip strength was measured alternately for both hands, During the test, each hand was measured 3 times with a 1-minute break between each measurement. NHANES used the maximum value of the respective measurements of both hands to obtain the value of the combined grip strength, which was expressed in kilograms.

### 2.4. Covariates

The selection of covariates was guided by previous literature identifying factors associated with CMI and skeletal muscle mass, and grip strength, to adjust for potential confounders in the relationship between CMI and muscle health.^[17–20]^ Demographic indicators included in this study include age, gender, race, education, and PIR. Lifestyle-related indicators covered smoking status, drinking status, physical activity (PA), and sedentary behavior. Smoking status was defined by asking whether participants had smoked more than 100 cigarettes cumulatively in their lifetime, and drinking status was determined by whether they had consumed more than 12 alcoholic drinks in the past year. PA and sedentary time were assessed using the Global PA Questionnaire and quantified using metabolic equivalent minutes. Height and weight were determined during the physical examination and subsequently categorized using the BMI into 3 groups: normal weight (BMI < 25 kg/m²), overweight (BMI 25–29.9 kg/m²), and obese (BMI ≥ 30 kg/m²). Health status variables, on the other hand, included hypertension, CVD, stroke, sleep disorders, and diabetes mellitus, and this information was obtained either through physician diagnosis or participant self-report. Specific measurements and detailed procedures for each of these variables are available through the official NHANES website (www.cdc.gov/nchs/nhanes/).

### 2.5. Statistical analysis

All statistical analyses followed Centers for Disease Control and Prevention guidelines and were based on NHANES sampling weights to accommodate a complex multistage stratified survey design. Continuous variables are presented as mean ± standard deviation, non-normally distributed data are presented as median and interquartile range (IQR), and categorical variables are presented as frequency (n) and percentage (%) Comparisons of CMI quartiles were performed using chi-square or Kruskal-Wallis H tests. Regression modeling assessed the association of CMI with muscle mass and grip strength using β coefficients and 95% confidence intervals (CIs), and 3 models were developed using smoothed curve fitting. Restricted cubic spline (RCS) models were used to explore nonlinear associations and threshold effects. Specifically, Model 1 was unadjusted; Model 2 adjusted for age, sex, race; and Model 3 further included education level, BMI, smoking status, drinking status, diabetes, hypertension, CVD, stroke, sleep disorders, PA, and sedentary time. To validate the assumptions of linear regression, we assessed the normality of residuals using the Shapiro–Wilk test and visual inspection of Q–Q plots. Homoscedasticity was evaluated using the Breusch–Pagan test. All assumptions were reasonably satisfied (*P* > .05). In addition, stratified analyses were performed based on age, gender, ethnicity, education level, BMI classification, smoking, alcohol consumption, and chronic disease history, and the robustness of the results was assessed by trend tests.^[21]^ To handle missing data, we conducted complete-case analysis and performed a sensitivity analysis using multiple imputation by chained equations. All statistical analyses were performed using R (version 4.4.1, http://www.R-project.org) and Empower Stats 4.2 (http://www.empowerstats.com).

## 3. Results

### 3.1. Basic characteristics of the study population

As shown in Table 1, 2719 subjects with a mean age of 38.7 ± 0.21 years, 52.02% males and 47.98% females were included in this study. They were categorized into 4 quartile groups according to CMI: Q1 (<0.26), Q2 (0.29–0.50), Q3 (0.50–0.86), and Q4 (>0.86). Mean skeletal muscle mass index (ASMI) was 7.898 kg/m² for all participants and increased with higher CMI quartiles (Q1: 7.0 [6.9–7.2]; Q2: 7.5 [7.4–7.7]; Q3: 8.1 [7.9–8.3]; Q4: 8.8 [8.6–8.9], *P* < .0001). Mean grip strength was 76.70 kg, again increasing with CMI (Q1: 71.5 [69.5–73.6]; Q4: 83.5 [81.8–85.1], *P* < .0001). Differences between quartiles were significant (*P* < .05) for age, gender, race, education level, BMI, smoking, diabetes, hypertension, coronary heart disease, sleep disorders, HDL cholesterol, and TG. Whereas, PIR, alcohol consumption, stroke, PA, and sedentary time were not statistically different between the 4 groups (*P* > .05).

**Table 1:** Weighted characteristics of the study population based on CMI quartiles.

Variables	Q1 (< 0.26)	Q2 (0.29–0.50)	Q3 (0.29–0.50)	Q4 (>0.86)	*P* value
Participants	680	679	680	680	
Age (yr)	34.0 (32.7–35.2)	37.6 (35.7–39.4)	39.9 (38.9–40.9)	41.5 (40.1–42.8)	<.0001
Gender (n, %)					<.0001
Male	265 (39.0%)	324 (47.7%)	351 (51.6%)	474 (69.7%)	
Female	415 (61.0%)	355 (52.3%)	329 (48.4%)	206 (30.3%)	
Race (n, %)					<.0001
Mexican American	44 (6.5%)	56 (8.3%)	84 (12.4%)	80 (11.7%)	
Other Hispanic	44 (6.4%)	35 (5.2%)	48 (7.0%)	50 (7.4%)	
Non-Hispanic White	396 (58.2%)	472 (69.5%)	419 (61.6%)	472 (69.4%)	
Non-Hispanic Black	113 (16.6%)	71 (10.5%)	69 (10.1%)	38 (5.6%)	
Other Races	84 (12.3%)	44 (6.5%)	61 (9.0%)	40 (5.9%)	
Education level (n, %)					<.0001
Less than junior high school	17 (2.5%)	18 (2.6%)	24 (3.5%)	34 (5.0%)	
Middle to high school	56 (8.2%)	63 (9.3%)	87 (12.8%)	95 (13.9%)	
High school graduate/GED or equivalent	121 (17.8%)	152 (22.4%)	136 (20.0%)	149 (21.9%)	
Some College or AA degree	223 (32.8%)	191 (28.1%)	237 (34.9%)	239 (35.1%)	
College Graduate or above	262 (38.6%)	256 (37.7%)	196 (28.8%)	163 (24.0%)	
PIR	2.9 (2.6–3.1)	3.0 (2.7–3.3)	2.8 (2.6–3.0)	2.7 (2.5–3.0)	.205
BMI (n, %)					<.0001
≤25	29 (4.2%)	117 (17.2%)	253 (37.2%)	543 (79.9%)	
>25, ≤30	161 (23.7%)	287 (42.3%)	299 (43.9%)	125 (18.4%)	
>30	490 (72.1%)	276 (40.6%)	129 (18.9%)	12 (1.7%)	
Smoking (n, %)					.0001
Yes	226 (33.3%)	244 (35.9%)	288 (42.3%)	335 (49.3%)	
No	454 (66.7%)	435 (64.1%)	392 (57.7%)	345 (50.7%)	
Drinking (n, %)					.194
Yes	538 (79.1%)	567 (83.5%)	544 (80.0%)	564 (83.0%)	
No	142 (20.9%)	112 (16.5%)	136 (20.0%)	116 (17.0%)	
Hypertension (n, %)					<.0001
Yes	83 (12.2%)	134 (19.7%)	171 (25.2%)	229 (33.7%)	
No	597 (87.8%)	545 (80.3%)	509 (74.8%)	451 (66.3%)	
CVD history (n, %)					.021
Yes	3 (0.4%)	8 (1.2%)	2 (0.3%)	14 (2.0%)	
No	677 (99.6%)	671 (98.8%)	678 (99.7%)	666 (98.0%)	
Stroke (n, %)					.687
Yes	7 (1.1%)	9 (1.3%)	12 (1.8%)	5 (0.8%)	
No	673 (98.9%)	670 (98.7%)	668 (98.2%)	675 (99.2%)	
Sleep disorder (n, %)					.003
Yes	32 (4.7%)	45 (6.7%)	64 (9.4%)	82 (12.0%)	
No	648 (95.3%)	634 (93.3%)	616 (90.6%)	598 (88.0%)	
Diabetes (n, %)					<.0001
Yes	12 (1.8%)	33 (4.9%)	42 (6.2%)	103 (15.1%)	
No	668 (98.2%)	646 (95.1%)	638 (93.8%)	577 (84.9%)	
HDL-C (mmol/L)	1.7 (1.7–1.8)	1.4 (1.4–1.5)	1.3 (1.2–1.3)	1.0 (1.0–1.0)	<.0001
TG (mmol/L)	0.6 (0.6–0.6)	0.9 (0.9–0.9)	1.3 (1.3–1.3)	2.7 (2.5–2.9)	<.0001
PA (MET·min/wk)	4871.2 (3152.1–6590.4)	4714.2 (3082.3–6346.1)	3878.5 (3083.8–4673.1)	3906.6 (3287.8–4525.4)	.654
Sedentary time (min/wk)	418.9 (357.0–480.8)	406.0 (385.6426.4)	386.1 (370.2402.1)	409.9 (392.4427.4)	.226
ASMI (kg/m²)	7.0 (6.9–7.2)	7.5 (7.4–7.7)	8.1 (7.9–8.3)	8.8 (8.6–8.9)	<.0001
Grip strength (kg)	71.5 (69.5–73.6)	75.2 (73.6–76.7)	76.7 (74.2–79.3)	83.5 (81.8–85.1)	<.0001

For continuous variables: survey-weighted mean (95% CI), *P*-value was by survey-weighted linear regression. For categorical variables: sample size (weighted percentage), *P*-value was by survey-weighted Chi-square test.

ASMI = Appendicular Skeletal Muscle Mass Index, BMI = body mass index, CMI = cardiometabolic index, CVD = cardiovascular disease, HDL-C = high-density lipoprotein cholesterol, MET = metabolic equivalent, PA = physical activity, PIR = the ratio of income to poverty, TG = triglyceride.

### 3.2. Association between CMI and muscle mass and grip strength

In this study, muscle mass was converted into a binary variable based on the thresholds of ASMI: < 7.0 kg/m² for men and < 5.5 kg/m² for women. However, data analysis revealed that only 11.55% of the population met the criteria for sarcopenia (Table S1, Supplemental Digital Content, https://links.lww.com/MD/Q747). Due to the small sample size, which did not meet the statistical requirements for weighted logistic regression analysis, a weighted generalized linear regression model was used to assess the relationship between CMI, muscle mass, and grip strength.

As shown in Table 2, Models 1 and 2 showed that CMI was significantly positively associated with skeletal muscle mass (Model 1: β = 0.38, 95% CI: 0.33–0.44; Model 2: β = 0.29, 95% CI: 0.25–0.34). However, in Model 3, the positive association remained but was attenuated (Model 3: β = 1.01, 95% CI: 0.86–1.17). After dividing CMI into quartiles, skeletal muscle mass significantly increased in the other 3 quartile groups (Q2, Q3, and Q4) compared to the lowest group (Q1), with statistically significant differences (*P* for trend < .000001). Regarding grip strength, Model 1 showed a significant positive association between CMI and grip strength (Model 1: β = 2.74, 95% CI: 2.02–3.46; *P* < .0001). This association was attenuated in Model 2 (Model 2: β = 0.86, 95% CI: 0.40–1.31; *P* = .0002). In the fully adjusted model, the association between CMI and grip strength regained significance (Model 3: β = 2.79, 95% CI: 1.10–4.47; *P* = .0012). Quartile analysis indicated that in Models 1 and 2, grip strength levels in the higher quartile groups (Q3 and Q4) were significantly higher than those in the lowest quartile group (Q1). However, in the fully adjusted model, this association disappeared, and the differences in grip strength levels between Q3, Q4, and Q1 were not statistically significant (*P* > .05).

**Table 2:** Association between CMI and skeletal muscle mass and grip strength.

	Model 1 β (95% CI) *P*-value	Model 2 β (95% CI) *P*-value	Model 3 β (95% CI) *P*-value
ASMI			
CMI (continuous)	0.38 (0.33 to 0.44) < .0001	0.29 (0.25 to 0.34) < .0001	1.01 (0.86 to 1.17) < .0001
CMI quartile			
Q1	Reference	Reference	Reference
Q2	0.48 (0.32 to 0.65) < .0001	0.42 (0.28 to 0.55) < .0001	0.24 (0.08 to 0.40) .0040
Q3	1.04 (0.87 to 1.21) < .0001	0.94 (0.81 to 1.08) < .0001	0.72 (0.54 to 0.90) < .0001
Q4	1.72 (1.55 to 1.89) < .0001	1.38 (1.24 to 1.53) < .0001	1.03 (0.80 to 1.25) < .0001
*P* for trend	<.000001	<.000001	<.000001
Grip strength			
CMI (continuous)	2.74 (2.02 to 3.46) < .0001	0.86 (0.40 to 1.31) .0002	2.79 (1.10 to 4.47) .0012
CMI quartile			
Q1	Reference	Reference	Reference
Q2	3.64 (1.32 to 5.96) .0021	1.15 (−0.31 to 2.62) .1234	−0.10 (−1.82 to 1.63) .9114
Q3	5.22 (2.87 to 7.56) < .0001	2.23 (0.73, 3.73) .0035	1.11 (−0.85 to 3.06) .2683
Q4	11.94 (9.62 to 14.26) < .0001	3.07 (1.53 to 4.61) < .0001	0.85 (−1.55 to 3.24) .4881
*P* for trend	<.00001	<.00001	<.00001

Model 1: Unadjusted. Model 2: Adjusted for sex, age, and race. Model 3: Adjusted for age, sex, race, education level, BMI, smoking status, alcohol consumption, diabetes, hypertension, coronary artery disease, stroke, sleep disorders, HDL-C, triglycerides, physical activity (PA), and sedentary time.

95% CI = 95% confidence interval, ASMI = Appendicular Skeletal Muscle Mass Index, CMI = Cardiometabolic Index, Q = quartile, β = effect size from linear regression.

### 3.3. Nonlinear relationship and threshold effect analysis

**Figure 2.**
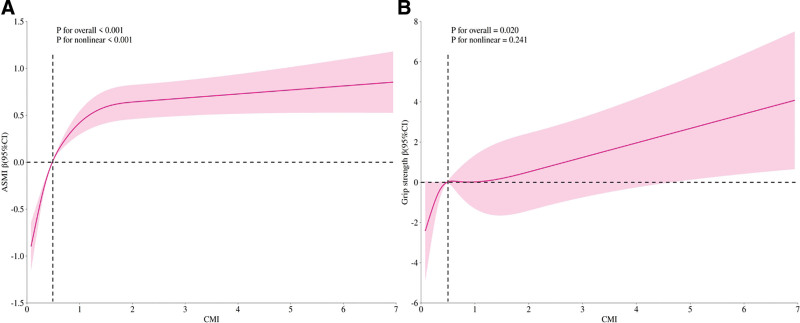
Dose–response relationship analysis. (A) Association between CMI and ASMI. (B) Association between CMI and grip strength. Adjustments were made for age(categorization), sex, race, education level, BMI (categorization), smoking status, Drinking status, diabetes, hypertension, CVD, stroke, sleep disorders, PA, and sedentary time. ASMI = Appendicular Skeletal Muscle Mass Index, CMI = Cardiometabolic Index, CVD = cardiovascular disease, PA = physical activity.

As shown in Figure 2, an RCS analysis was performed to further explore potential nonlinear relationships. The results indicated a significant nonlinear relationship between CMI and ASMI (*P* for nonlinearity < .001). As shown in Table 3, the association between CMI and ASMI was stronger when CMI was below 0.6 (β = 5.09, 95% CI: 4.57–5.61), and the effect became less pronounced above this threshold (β = 1.46, 95% CI: 1.30–1.62). The log-likelihood ratio test supported a significant threshold effect (*P* < .001). In contrast, the relationship between CMI and grip strength was approximately linear, and no threshold was identified.

**Table 3:** Threshold effect analysis.

CMI	β (95% CI) *P* value
ASMI	
Standard linear model	1.02 (0.86–1.17) < .0001
CMI < 0.6	5.09 (4.57–5.61) < .0001
CMI > 0.6	1.46 (1.30–1.62) < .0001
Log-likelihood ratio test	<.001

Adjustments were made for age(categorization), sex, race, education level, BMI (categorization), smoking status, drinking status, diabetes, hypertension, CVD, stroke, sleep disorders, PA, and sedentary time.

95% CI = 95% Confidence Interval, ASMI = Appendicular Skeletal Muscle Mass Index, CMI = Cardiometabolic Index, CVD = cardiovascular disease, β = effect size from linear regression.

### 3.4. Subgroup analysis

**Figure 3.**
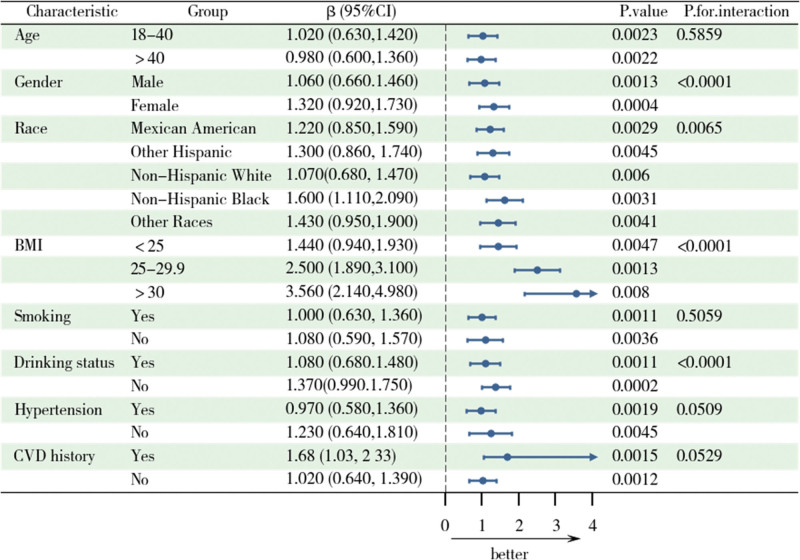
Forest plot and subgroup analysis of CMI associated with ASMI. Adjustments were made for age, sex, race, education level, BMI, smoking status, Drinking status, diabetes, hypertension, CVD, stroke, sleep disorders, PA, and sedentary time. ASMI = Appendicular Skeletal Muscle Mass Index, BMI = body mass index, CMI = Cardiometabolic Index, CVD = cardiovascular disease, PA = physical activity.

Figure 3 illustrates the subgroup analysis and interaction test of the relationship between CMI and muscle mass, which, after adjusting for confounders, showed a positive association between CMI and muscle mass. The association differed significantly by gender, race, BMI, and drinking status (interaction *P* < .05), with stronger associations among women, non-Hispanic blacks, those with higher BMI, and nondrinkers. In addition, the interaction *P* values for the hypertension and CVD groups approached significance (*P* = .0509 and .0529), suggesting that these factors may influence the relationship between CMI and muscle mass.

**Figure 4.**
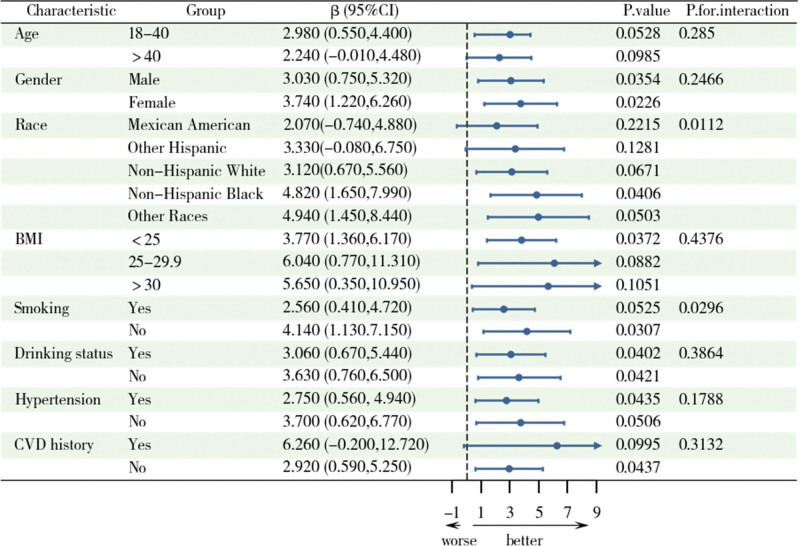
Forest plot and subgroup analysis of CMI associated with Grip strength. Adjustments were made for age, sex, race, education level, BMI, smoking status, Drinking status, diabetes, hypertension, CVD, stroke, sleep disorders, PA, and sedentary time. BMI = body mass index, CMI = Cardiometabolic Index, CVD = cardiovascular disease.

Figure 4 shows an overall positive correlation between CMI and grip strength, with variations across different subgroups. Significant interactions were observed for race (*P* = .0112) and smoking status (*P* = .0296), indicating that these factors significantly influence the relationship between CMI and grip strength. The association was more pronounced among nonsmokers, non-Hispanic Black individuals, and those of other races. However, differences across age, sex, alcohol consumption status, hypertension, and CVD history were not statistically significant.

### 3.5. Sensitivity and additional analyses

Sensitivity analyses were conducted to confirm the consistency and robustness of the study. After excluding lifestyle variables, the effect of CMI on ASMI remained direct and robust, whereas the association with grip strength weakened significantly, suggesting that CMI’s impact on grip strength is more susceptible to external factors (Table S2, Supplemental Digital Content, https://links.lww.com/MD/Q747). After excluding health-related variables, the association between CMI and ASMI remained robust. In contrast, the association between CMI and grip strength significantly weakened (Table S3, Supplemental Digital Content, https://links.lww.com/MD/Q747). We also explored the association between CMI and ALM in lean body mass in the extremities (Table S4, Supplemental Digital Content, https://links.lww.com/MD/Q747) and found that CMI was significantly and positively associated with ALM, which was significantly higher in the high CMI group than in the low CMI group. Another indicator of muscle mass, ALM and body mass index ratio (ALM/BMI; Table S5, Supplemental Digital Content, https://links.lww.com/MD/Q747), was used to investigate the relationship between CMI and ALM/BMI. We found that with an increase in CMI, ALM/BMI significantly decreased, indicating that individuals with higher CMI have a lower proportion of lean mass relative to BMI. In addition, we used multiple interpolation methods (multiple imputation by chained equations) to confirm the robustness of the association between CMI and ASMI (Table S6, Supplemental Digital Content, https://links.lww.com/MD/Q747).

## 4. Discussion

In this cross-sectional study, a nationally representative population sample was selected to assess the correlation between CMI and ASMI and muscle strength. The results showed a significant positive trend of association between CMI and ASMI and strength. With an increase in the CMI quartiles, both skeletal muscle mass, and grip strength levels gradually increased. However, after full adjustment, the association between CMI and grip strength weakened and became nonsignificant, while the positive correlation with skeletal muscle mass remained. Furthermore, RCS analysis revealed a nonlinear relationship between CMI and skeletal muscle mass. The nonlinear analysis suggested a threshold effect: at lower levels (<0.6), CMI had the strongest promoting effect on muscle mass, whereas at higher levels (>0.6), this effect significantly diminished. In contrast, the relationship between CMI and grip strength is more complex. Although a positive correlation was observed in the preliminary model, this relationship became nonsignificant after full adjustment. Subgroup analysis further revealed that factors such as sex, race, BMI, and lifestyle significantly influenced the relationship between CMI and skeletal muscle health.

Previous studies mainly focused on the relationship between single metabolic indicators (such as BMI, WC, or lipid levels) and skeletal muscle mass.^[22]^ Some studies have shown that larger WC or higher triglyceride levels are significantly associated with the risk of sarcopenia.^[23,24]^ In contrast, CMI, as a comprehensive metabolic health index,^[12]^ can more comprehensively reflect fat distribution and metabolic burden, thus providing a more accurate understanding of the impact of metabolic health on skeletal muscle mass. This study fills this research gap and further demonstrates the potential value of CMI as a predictor of muscle health. The positive correlation between CMI and grip strength was significant in the preliminary model, but weakened and lost statistical significance after full adjustment. Consistent with previous studies, grip strength is not only influenced by metabolic health but may also be closely associated with various external factors, such as PA level, diet quality, mental state, and inflammation levels.^[25,26]^ Furthermore, studies have shown that age-related declines in muscle strength often exhibit complex nonlinear changes, which may further obscure the direct association between CMI and grip strength.^[27]^ The threshold effect reveals that at lower CMI levels, its impact on skeletal muscle mass is more pronounced, whereas at higher CMI levels, this effect diminishes or even reverses. This may be related to the excessive metabolic burden in individuals with higher CMI levels, particularly the negative effects of inflammation, insulin resistance, and oxidative stress due to fat accumulation, which may impair skeletal muscle function.^[28,29]^ Subgroup analysis showed that sex, race, and BMI significantly modulate the relationship between CMI and muscle health. For example, non-Hispanic blacks have stronger skeletal muscle mass performance at higher CMI,^[30]^ which may be related to their higher muscle mass base and metabolic adaptations.^[14]^ In women and individuals with higher BMI, the positive correlation between CMI and skeletal muscle mass was more significant, suggesting that improvements in metabolic health may have greater potential benefits for muscle health in these groups.

CMI reflects the extent of lipid metabolism disorders,^[31]^ and lipid metabolism dysregulation (e.g., elevated TG and reduced HDL-C) may have a complex interaction with skeletal muscle function.^[32]^ Excessive accumulation of TG and adipose tissue can inhibit protein synthesis through the release of pro-inflammatory cytokines (e.g., tumor necrosis factor-alpha and interleukin-6), leading to a decline in skeletal muscle mass.^[33,34]^ Insulin resistance is a common characteristic of individuals with higher CMI levels, which may further affect muscle mass by inhibiting skeletal muscle glucose uptake and reducing the activation of anabolic signaling pathways (e.g., PI3K/AKT/mTOR pathway).^[35,36]^ The complexity of metabolic inflammation and muscle strength may be related to the level of metabolic inflammation.^[37]^ Individuals with higher CMI levels may have elevated systemic inflammation, which not only affects muscle mass but may also negatively impact muscle strength through a decline in neuromuscular junction function.^[38]^ This study emphasizes the potential clinical value of CMI as a tool for assessing metabolic health. Monitoring CMI can not only help predict the risk of CVD and metabolic disorders but may also serve as an effective indicator for assessing skeletal muscle mass, particularly in the elderly or populations at high risk for metabolic disorders. Our findings suggest that CMI may serve as a simple and practical indicator for early identification of individuals at risk of poor muscle strength. However, prospective studies are warranted to determine whether interventions targeting CMI can help preserve musculoskeletal function in aging populations. Furthermore, the nonlinear effects of CMI suggest that personalized interventions based on different CMI levels (such as controlling body fat, optimizing lipid metabolism, and managing inflammation) may be crucial for maintaining muscle health.

In conclusion, our findings demonstrate a significant and complex relationship between CMI and skeletal muscle health, especially muscle mass. This study highlights the clinical utility of CMI as a simple yet informative indicator for evaluating both metabolic and musculoskeletal status, offering valuable guidance for early screening and integrated health management in metabolic-risk populations.

## 5. Limitations

The present study has several limitations. First, due to its cross-sectional design, the study could not establish causality or address the potential bidirectional relationship between CMI and muscle mass and strength. While our findings provide meaningful associations, they do not imply temporal or causal directions. Second, our data were exclusively derived from NHANES 2011–2014 cycles, primarily because comprehensive measures of ASMI and grip strength were uniquely available during these periods. This limited availability prevented longitudinal analysis of changes over time. Although longitudinal data were not used in this study, future research may incorporate cohorts such as the English Longitudinal Study of Ageing (ELSA) and the China Health and Retirement Longitudinal Study (CHARLS) to clarify temporal relationships. Finally, as our sample was drawn from the U.S. population, the generalizability of findings to other populations with different racial or cultural backgrounds may be limited. Future studies using international longitudinal cohorts are encouraged to improve the applicability of the findings.

## 6. Conclusion

CMI is significantly positively correlated with skeletal muscle mass, and this association exhibits nonlinear and threshold effects. The relationship between CMI and muscle strength is more complex and may be influenced by other confounding factors.

## Acknowledgments

The authors gratefully acknowledge the National Center for Health Statistics (NCHS) for providing access to the publicly available NHANES datasets.

## Author contributions

**Conceptualization:** Shiqi Yu.

**Data curation:** Shiqi Yu, Young-Je Sim, Han Yuan.

**Formal analysis:** Kunyi Huang.

**Investigation:** Han Yuan.

**Methodology:** Yang Wang.

**Software:** Chen Chen, Zhenhao Lin.

**Validation:** Yuwen Shangguan.

**Visualization:** Yuwen Shangguan, Young-Je Sim.

**Writing – original draft:** Yuwen Shangguan.

**Writing – review & editing:** Yuwen Shangguan, Zining Zhu.

## Supplementary Material


