# Supplementary material for: Association between Cardiometabolic Index (CMI) with muscle mass and strength in US adults: A cross-sectional study
Source: Medicine (Baltimore). 2025 Nov 21;104(47):e46128. doi: 10.1097/MD.0000000000046128 (PMC12643643; doi:10.1097/MD.0000000000046128)
Supplement: Supplementary file 1 [file medi-104-e46128-s001.docx]

**Association between cardiometabolic index CMI with muscle mass and strength in US adults: a cross-sectional study**

Yuwen ShangGuan, PhD: First author, Department of Exercise Physiology, Kunsan National University, Gunsan 541150, Jeollabuk-do, South Korea

**Supplementary Table S1.** Distribution of Sarcopenia Population

| Sarcopenia | 1 | 2 | P-value |
| --- | --- | --- | --- |
| N (%) | 314(11.55%) | 2405(88.45%) |  |
| ASMI | 5.70 ± 0.81 | 8.19 ± 1.58 | <0.001 |

1 indicates ASMI < 7.0 kg/m² for men or < 5.5 kg/m² for women.

2 indicates ASMI ≥ 7.0 kg/m² for men or ≥ 5.5 kg/m² for women.

**Supplementary Table S2.** Exclusion of lifestyle variables: smoking status, alcohol consumption, physical activity (PA), and sedentary time.

|  | Model 1 β (95% Cl)  p-value | Model 2 β (95% Cl)  p-value | Model 3 β (95% Cl)  p-value |
| --- | --- | --- | --- |
| ASMI |  |  |  |
| CMI | 0.38 (0.33, 0.44) <0.0001 | 0.29 (0.25, 0.34) <0.0001 | 1.03 (0.87, 1.18) <0.0001 |
| CMI quartile |  |  |  |
| Q1 | Reference | Reference | Reference |
| Q2 | 0.48 (0.32, 0.65) <0.0001 | 0.42 (0.28, 0.55) <0.0001 | 0.22 (0.06, 0.38) 0.0062 |
| Q3 | 1.04 (0.87, 1.21) <0.0001 | 0.94 (0.81, 1.08) <0.0001 | 0.69 (0.51, 0.87) <0.0001 |
| Q4 | 1.72 (1.55, 1.89) <0.0001 | 1.38 (1.24, 1.53) <0.0001 | 0.97 (0.75, 1.19) <0.0001 |
| Grip strength |  |  |  |
| CMI | 2.74 (2.02, 3.46) <0.0001 | 0.86 (0.40, 1.31) 0.0002 | 2.75 (1.08, 4.43) 0.0013 |
| CMI quartile |  |  |  |
| Q1 | Reference | Reference | Reference |
| Q2 | 3.64 (1.32, 5.96) 0.0021 | 1.15 (-0.31, 2.62) 0.1234 | -0.09 (-1.79, 1.60) 0.9154 |
| Q3 | 5.22 (2.87, 7.56) <0.0001 | 2.23 (0.73, 3.73) 0.0035 | 0.91 (-1.00, 2.83) 0.3503 |
| Q4 | 11.94 (9.62, 14.26) <0.0001 | 3.07 (1.53, 4.61) <0.0001 | 0.48 (-1.88, 2.83) 0.6918 |

CMI: Cardiometabolic Index; Q: Quartile; β: Effect size from linear regression; 95% CI: 95% Confidence Interval. Model 1: Unadjusted for covariates. Model 2: Adjusted for sex, age, and race. Model 3: Adjusted for age, sex, race, education level, BMI, diabetes, hypertension, CVD, stroke, sleep disorders.

**Supplementary Table S3.** Exclusion of health-related variables: hypertension, stroke, diabetes, coronary heart disease, and sleep disorders.

|  | Model 1 β (95% Cl)  p-value | Model 2 β (95% Cl)  p-value | Model 3 β (95% Cl)  p-value |
| --- | --- | --- | --- |
| ASMI |  |  |  |
| CMI | 0.38 (0.33, 0.44) <0.0001 | 0.29 (0.25, 0.34) <0.0001 | 1.12 (0.96, 1.28) <0.0001 |
| CMI quartile |  |  |  |
| Q1 | Reference | Reference | Reference |
| Q2 | 0.48 (0.32, 0.65) <0.0001 | 0.42 (0.28, 0.55) <0.0001 | 0.26 (0.09, 0.42) 0.0021 |
| Q3 | 1.04 (0.87, 1.21) <0.0001 | 0.94 (0.81, 1.08) <0.0001 | 0.76 (0.57, 0.94) <0.0001 |
| Q4 | 1.72 (1.55, 1.89) <0.0001 | 1.38 (1.24, 1.53) <0.0001 | 1.12 (0.89, 1.34) <0.0001 |
| Grip strength |  |  |  |
| CMI | 2.74 (2.02, 3.46) <0.0001 | 0.86 (0.40, 1.31) 0.0002 | 2.50 (0.83, 4.18) 0.0034 |
| CMI quartile |  |  |  |
| Q1 | Reference | Reference | Reference |
| Q2 | 3.64 (1.32, 5.96) 0.0021 | 1.15 (-0.31, 2.62) 0.1234 | -0.06 (-1.79, 1.68) 0.9501 |
| Q3 | 5.22 (2.87, 7.56) <0.0001 | 2.23 (0.73, 3.73) 0.0035 | 1.22 (-0.74, 3.18) 0.2221 |
| Q4 | 11.94 (9.62, 14.26) <0.0001 | 3.07 (1.53, 4.61) <0.0001 | 0.90 (-1.49, 3.30) 0.4586 |

CMI: Cardiometabolic Index; Q: Quartile; β: Effect size from linear regression; 95% CI: 95% Confidence Interval. Model 1: Unadjusted for covariates. Model 2: Adjusted for sex, age, and race. Model 3: Adjusted for age, sex, race, education level, BMI, smoking status, alcohol consumption, PA, and sedentary time.

**Supplementary Table S4.** Association Analysis of CMI and Muscle Mass (ALM/BMI)

|  | Model 1 β (95% Cl)  p-value | Model 2 β (95% Cl)  p-value | Model 3 β (95% Cl)  p-value |
| --- | --- | --- | --- |
| Skeletal muscle mass（ALM/BMI） | | | |
| CMI(continuous) | 0.00 (-0.01, 0.01) 0.9347 | -0.02 (-0.02, -0.01) <0.0001 | -0.04 (-0.05, -0.03) <0.0001 |
| CMI (quartile) |  | | |
| Q1 | Reference | Reference | Reference |
| Q2 | -0.02 (-0.04, 0.00) 0.0587 | -0.04 (-0.05, -0.03) <0.0001 | -0.05 (-0.07, -0.04) <0.0001 |
| Q3 | -0.05 (-0.07, -0.02) <0.0001 | -0.07 (-0.08, -0.06) <0.0001 | -0.08 (-0.10, -0.07) <0.0001 |
| Q4 | -0.02 (-0.05, -0.00) 0.0298 | -0.11 (-0.12, -0.09) <0.0001 | -0.13 (-0.14, -0.11) <0.0001 |
| P for trend | 0.029062 | 0.029062 | 0.029062 |

Model 1: Unadjusted for covariates. Model 2: Adjusted for sex, age, and race. Model 3: Adjusted for age, sex, race, education level, BMI, smoking status, alcohol consumption, diabetes, hypertension, coronary heart disease, stroke, sleep disorders, physical activity (PA), and sedentary time.

**Supplementary Table S5.** Association Analysis of CMI and ALM

|  | Model 1 β (95% Cl)  p-value | Model 2 β (95% Cl)  p-value | Model 3 β (95% Cl)  p-value |
| --- | --- | --- | --- |
| CMI | 1.30 (1.09, 1.50) <0.0001 | 0.82 (0.68, 0.97) <0.0001 | 2.81 (2.29, 3.32) <0.0001 |
| CMI quartile |  |  |  |
| Q1 | Reference | Reference | Reference |
| Q2 | 1.84 (1.19, 2.49) <0.0001 | 1.30 (0.84, 1.75) <0.0001 | 0.55 (0.01, 1.09) 0.0446 |
| Q3 | 3.24 (2.59, 3.90) <0.0001 | 2.59 (2.13, 3.06) <0.0001 | 1.69 (1.08, 2.30) <0.0001 |
| Q4 | 5.85 (5.20, 6.49) <0.0001 | 3.82 (3.34, 4.30) <0.0001 | 2.39 (1.64, 3.13) <0.0001 |
| P for trend | <0.000001 | <0.000001 | <0.000001 |

Model 1: Unadjusted. Model 2: Adjusted for sex, age, and race. Model 3: Adjusted for age, sex, race, education level, BMI, smoking status, drinking status, diabetes, hypertension, CVD, stroke, sleep disorders, PA, and sedentary time.

**Supplementary Table S6.** Sensitivity Analysis of the Association Between Cardiometabolic Index (CMI) and Skeletal Muscle Mass and Grip Strength After Multiple Imputation Using Chained Equations (MICE)

|  | Model 1 β (95% Cl)  p-value | Model 2 β (95% Cl)  p-value | Model 3 β (95% Cl)  p-value |
| --- | --- | --- | --- |
| ASMI | | | |
| CMI(continuous) | 0.36（0.32, 0.41） <0.0001 | 0.28（0.24, 0.32） <0.0001 | 0.98（0.84，1.12） <0.0001 |
| CMI quartile |  | | |
| Q1 | Reference | Reference | Reference |
| Q2 | 0.45 (0.30, 0.60) <0.0001 | 0.39（0.26，0.52） <0.0001 | 0.22（0.07，0.37）0.0035 |
| Q3 | 0.98（0.83，1.13） <0.0001 | 0.89 (0.76, 1.02) <0.0001 | 0.68 (0.52, 0.86) <0.0001 |
| Q4 | 1.70 (1.52, 1.87) <0.0001 | 1.33 (1.20, 1.46) <0.0001 | 0.96 (0.76, 1.16) <0.0001 |
| P for trend | <0.000001 | <0.000001 | <0.000001 |
| Grip strength | | | |
| CMI(continuous) | 2.70(2.01,3.35) <0.0001 | 0.82 (0.39, 1.25) 0.0002 | 2.65 (1.05, 4.25) 0.0012 |
| CMI quartile |  | | |
| Q1 | Reference | Reference | Reference |
| Q2 | 3.53 (1.28, 5.76) 0.0021 | 1.08 (-0.28, 2.44) 0.1198 | -0.11 (-1.83, 1.63) 0.9114 |
| Q3 | 5.08 (2.91, 7.25) <0.0001 | 2.15 (0.72, 3.58) 0.0032 | 1.02 (-0.85, 2.89) 0.2845 |
| Q4 | 11.76 (9.52, 14.00) <0.0001 | 2.98 (1.50, 4.46) <0.0001 | 0.78 (-1.48, 3.04) 0.4985 |
| P for trend | <0.00001 | <0.00001 | <0.00001 |

Model 1: Unadjusted. Model 2: Adjusted for sex, age, and race. Model 3: Adjusted for age, sex, race, education level, BMI, smoking status, drinking status, diabetes, hypertension, CVD, stroke, sleep disorders, PA, and sedentary time.
